# Assessment of Time-to-Treatment Initiation and Survival in a Cohort of Patients With Common Cancers

**DOI:** 10.1001/jamanetworkopen.2020.30072

**Published:** 2020-12-14

**Authors:** Eugene B. Cone, Maya Marchese, Marco Paciotti, David-Dan Nguyen, Junaid Nabi, Alexander P. Cole, George Molina, Rose L. Molina, Christina A. Minami, Lorelei A. Mucci, Adam S. Kibel, Quoc-Dien Trinh

**Affiliations:** 1 Division of Urological Surgery, Brigham and Women’s Hospital, Boston, Massachusetts; 2 Center for Surgery and Public Health, Brigham and Women’s Hospital, Boston, Massachusetts; 3 Department of Urology, Humanitas Clinical and Research Center IRCCS, Rozzano, Italy; 4 Division of Surgical Oncology, Brigham and Women’s Hospital, Boston, Massachusetts; 5 Department of Obstetrics and Gynecology, Beth Israel Deaconess Medical Center, Boston, Massachusetts; 6 Division of Breast Surgery, Brigham and Women’s Hospital, Boston, Massachusetts; 7 Department of Epidemiology, Harvard T.H. Chan School of Public Health, Boston, Massachusetts

## Abstract

**Importance:**

Resource limitations because of pandemic or other stresses on infrastructure necessitate the triage of time-sensitive care, including cancer treatments. Optimal time to treatment is underexplored, so recommendations for which cancer treatments can be deferred are often based on expert opinion.

**Objective:**

To evaluate the association between increased time to definitive therapy and mortality as a function of cancer type and stage for the 4 most prevalent cancers in the US.

**Design, Setting, and Participants:**

This cohort study assessed treatment and outcome information from patients with nonmetastatic breast, prostate, non-small cell lung (NSCLC), and colon cancers from 2004 to 2015, with data analyzed January to March 2020. Data on outcomes associated with appropriate curative-intent surgical, radiation, or medical therapy were gathered from the National Cancer Database.

**Exposures:**

Time-to-treatment initiation (TTI), the interval between diagnosis and therapy, using intervals of 8 to 60, 61 to 120, 121 to 180, and greater than 180 days.

**Main Outcomes and Measures:**

5-year and 10-year predicted all-cause mortality.

**Results:**

This study included 2 241 706 patients (mean [SD] age 63 [11.9] years, 1 268 794 [56.6%] women, 1 880 317 [83.9%] White): 1 165 585 (52.0%) with breast cancer, 853 030 (38.1%) with prostate cancer, 130 597 (5.8%) with NSCLC, and 92 494 (4.1%) with colon cancer. Median (interquartile range) TTI by cancer was 32 (21-48) days for breast, 79 (55-117) days for prostate, 41 (27-62) days for NSCLC, and 26 (16-40) days for colon. Across all cancers, a general increase in the 5-year and 10-year predicted mortality was associated with increasing TTI. The most pronounced mortality association was for colon cancer (eg, 5 y predicted mortality, stage III: TTI 61-120 d, 38.9% vs. 181-365 d, 47.8%), followed by stage I NSCLC (5 y predicted mortality: TTI 61-120 d, 47.4% vs 181-365 d, 47.6%), while survival for prostate cancer was least associated (eg, 5 y predicted mortality, high risk: TTI 61-120 d, 12.8% vs 181-365 d, 14.1%), followed by breast cancer (eg, 5 y predicted mortality, stage I: TTI 61-120 d, 11.0% vs. 181-365 d, 15.2%). A nonsignificant difference in treatment delays and worsened survival was observed for stage II lung cancer patients—who had the highest all-cause mortality for any TTI regardless of treatment timing.

**Conclusions and Relevance:**

In this cohort study, for all studied cancers there was evidence that shorter TTI was associated with lower mortality, suggesting an indirect association between treatment deferral and mortality that may not become evident for years. In contrast to current pandemic-related guidelines, these findings support more timely definitive treatment for intermediate-risk and high-risk prostate cancer.

## Key Points

**Question:**

What is the association between delays in treatment initiation for common cancers, as are necessary in resource-limited settings and pandemic conditions, with mortality?

**Findings:**

In this cohort study including 2 241 706 patients with breast, prostate, non-small cell lung, and colon cancer, generally higher all-cause mortality was associated with increasing time to treatment, although the degree varied by cancer type and stage. Patients with colon and lung cancer had the highest mortality associated with increased time to treatment.

**Meaning:**

These findings emphasize the importance of timely cancer treatment, and, in contrast to current pandemic-related guidelines, support more prompt definitive treatment for intermediate-risk and high-risk prostate cancer.

## Teaser

This cohort study of patients with breast, prostate, non-small cell lung, and colon cancer assesses the association of time from diagnosis to treatment with all-cause mortality.

## Introduction

The coronavirus disease 2019 (COVID-19) pandemic virus has overwhelmed health care systems, forcing redistribution of supplies, personnel, and treatments.^1,2,3^ To efficiently utilize scarce resources while minimizing exposure risk to patients and staff, oncologists have been urged to triage cancer care.^4^ Such deferral of care is difficult to implement as cancer remains a leading cause of death^5^ and early intervention can improve patient outcomes.^6^

Lengthy time to treatment initiation (TTI—the period between diagnosis and the start of definitive treatment) has previously been associated with an absolute increased risk of mortality, from 1.2% to 3.2% per week of delayed treatment in cancers such as lung, kidney, and pancreas,^7^ with inconsistent effects over time.^8^ For breast cancer, increasing TTI has been associated with worse survival, especially for underserved patients.^9,10,11^ For head and neck cancers, TTI beyond 2 months was associated with a 3-fold increase in mortality.^12,13,14,15^ A systematic review of symptomatic cancers found some association between shorter TTI and better clinical outcomes (primarily survival) for breast, colorectal, head and neck, and testicular cancers, but the findings were limited because of poor quality studies and heterogeneous methodology.^6^ As such, triaging cancer patients presents implementation challenges as there are no preexisting guidelines on optimal TTI across diseases, and guidelines released in response to the COVID pandemic were largely based on expert opinion.^4,16^

Understanding the impact of TTI on survival is crucial in allocating resources efficiently, especially when unforeseeable limitations arise—either because of external pressures such as pandemics, or internal factors such as underfunding, understaffing, or supply chain constraints. We therefore evaluated the association between increasing time to definitive therapy and overall survival as a function of cancer type, stage, and treatment modality for patients treated with curative intent for nonmetastatic disease in the 4 most prevalent cancers in the US: breast cancer, prostate cancer, non-small cell lung cancer (NSCLC), and colon cancer.^17^ We hypothesized that increased TTI would be negatively associated with overall survival for all cancers and that the effect size would vary by cancer type and stage. As we only focused on National Comprehensive Cancer Network (NCCN) guideline-approved treatments, we assumed that outcomes would not vary by treatment modality.

## Methods

### Data Source

We used the National Cancer Database (NCDB), a nationwide oncology-specific database administered by the Commission on Cancer and the American Cancer Society. The NCDB captures over 70% of cancer diagnoses in the US, including over 29 million individual cancers from over 1500 accredited facilities, which are abstracted by trained reviewers.^18,19^ This research was approved by the Partners Healthcare institutional review board, and was exempted from informed consent requirements because it used deidentified data from NCDB files; the manuscript reporting our findings was written in adherence with the Strengthening the Reporting of Observational Studies in Epidemiology (STROBE) reporting guideline.

### Patient Population

From January through March 2020, using NCDB years 2004 to 2015, we identified patients diagnosed with nonmetastatic breast cancer, prostate cancer, NSCLC, and colon cancer at stages eligible for definitive therapy per era-appropriate NCCN guidelines (complete eligibility criteria in eAppendix 1 in the Supplement). These cancers were selected as they are the 4 most common US cancers, they span a range of clinical aggression, they are treated in most accredited cancer facilities, and patients are eligible for definitive therapy via a variety of modalities.^18,20^ Stage III lung cancer was not included as there is no validated NCDB method to determine patient eligibility for definitive therapy. Prostate cancer was defined by NCCN risk category per standard of practice in the US. Stage 0 colon cancer was excluded as most patients are adequately treated via diagnostic polyp removal.

### Exposure

The exposure was TTI—the time in days from the date of diagnosis to receipt of definitive therapy for the patient’s disease. Receipt of definitive therapy was defined for each cancer subtype using 2004 to 2015 era-appropriate NCCN guidelines reviewed by cancer-specific disease experts (complete eligibility criteria in eAppendix 2 in the Supplement). TTI was a categorical variable with 4 levels: 8 to 60, 61 to 120, 121 to 180, and greater than 180 days. These time periods were chosen a priori as clinically useful and reflective of practice patterns. For example, a decision to defer all treatment for a given cancer and stage for a 2-month period during a pandemic is more readily implemented and impactful than a decision to stop treatment for 1 or 2 weeks on a hospital level. Patients with TTI within 1 week of diagnosis were excluded, as this may not represent elective surgery, as were those with TTI greater than 1 year because of the small sample sizes and validity concerns.

### Variables of Interest

We abstracted clinical demographic variables, which included age at diagnosis, race (as defined by NCDB), sex, facility type, facility county, county-level median household income, residence type, county-level median education level, diagnosis year, insurance status, and Charlson-Deyo score to be used as model covariates. Tumor-specific variables were also collected, including American Joint Committee on Cancer clinical stage (*AJCC Cancer Staging Manual*, *Eighth Edition*), grade, histology (where appropriate), and receptor status (where appropriate). Modality of any treatment received was abstracted (ie, surgery, chemotherapy, radiation therapy) and used to define definitive therapy, as was time from biopsy-based diagnosis to receipt of treatments. Given disparities in both preexisting cancer outcomes and the impact of the pandemic on racial minority groups,^21,22^ we also examined whether the association between TTI and predicted mortality varied by race.

### Outcome Measure

The primary outcome was all-cause mortality. The estimates of interest were predicted 5-year and 10-year all-cause mortality. We accepted as a limitation that we would not be able to identify the reason for delay, and that this could introduce bias.

### Statistical Analysis

To assess the association between TTI and predicted all-cause mortality for each cancer type, we fit a Cox proportional hazards model for each cancer and stage (assuming each stage represented separate populations) with TTI as the predictor, adjusting for the aforementioned covariates and clustering standard errors by facility, while testing to ensure proportional hazards assumptions were met.^23^ We tested for differences in TTI hazards, using the 8-to-60–day TTI group as referent and testing each 60-day cohort against it. We then used each model’s estimated log hazard ratio coefficients to compute mean predicted mortality outcomes for patients in each level of TTI by taking the linear combination of the model estimates set at each level of TTI and time and the mean of all covariates. Deriving predicted outcomes, while setting the covariate values at their mean, allowed us to estimate the mean difference in outcomes for each patient under different TTI while conserving their clinical and demographic characteristics. The mean predicted outcomes for each time point–TTI combination were then transformed to the predicted probability of mortality at that TTI and time point. We then extracted the 5-year and 10-year predicted mortality probabilities for each TTI. Predicted mortality was used, based on model estimates taken from the actual mortality data of the cohort within the NCDB, to provide a smoothed curve as a continuous function of time that could be easily interpreted by clinicians and policymakers. We accepted that groups with small sample sizes would yield less precise estimates but countered this by choosing highly incident cancers yielding large cohort sizes.

## Results

**Figure 1.**
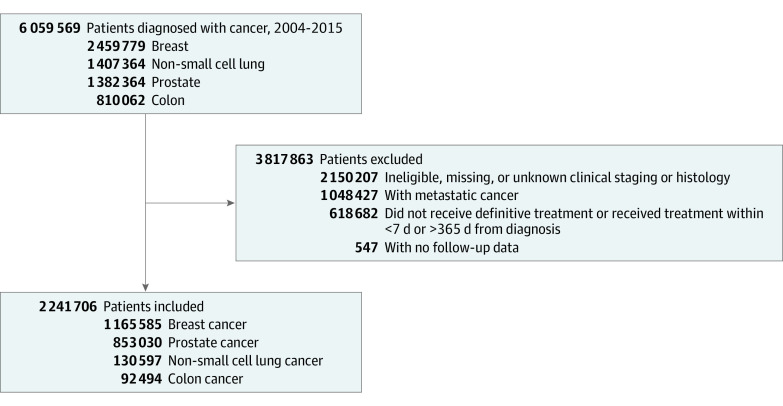
Flowchart of Patient Selection for Analysis

We included 2 241 706 patients (mean [SD] age of 63 [11.9]; 1 268 794 [56.6%] women; and 1 880 317 [83.9%] White) (Figure 1). By disease, 1 165 585 (52.0%) patients had breast cancer, 853 030 (38.1%) had prostate cancer, 130 597 (5.8%) had NSCLC, and 92 494 (4.1%) had colon cancer. Full clinical demographic characteristics can be found in Table 1. Median (interquartile range [IQR]) TTI was 32 (21-48) days for breast, 79 (55-117) days for prostate, 41 (27-62) days for NSCLC, and 26 (16-40) days for colon (medians with IQRs by cancer with stage can be found in Table 2).

**Table 1.** Baseline Clinical Demographic Characteristics of Nonmetastatic Cancer Patients in NCDB Database, 2004-2015

Characteristics	Patients, No. (%)			
	Breast (n = 1 165 585)	Colon (n = 92 494)	NSC lung (n = 130 597)	Prostate (n = 853 030)
Age, mean (SD), y	61.0 (13.3)	68.2 (12.5)	68.4 (9.5)	64.2 (8.2)
Sex				
Men	9975 (0.9)	45 690 (49.4)	64 217 (49.2)	853 030 (100)
Women	1 155 610 (99.1)	46 804 (50.6)	66 380 (50.8)	0
Race				
White	983 914 (84.4)	78 371 (84.7)	115 707 (88.6)	702 325 (82.3)
Black	123 602 (10.6)	10 105 (10.9)	10 672 (8.2)	114 866 (13.5)
Other	47 839 (4.1)	3311 (3.6)	3428 (2.6)	22 492 (2.6)
Unknown	10 230 (0.9)	707 (0.8)	790 (0.6)	13 347 (1.6)
Charlson-Deyo comorbidity index				
0	972 709 (83.5)	62 598 (67.7)	65 501 (50.2)	714 779 (83.8)
1	154 624 (13.3)	21 251 (23.0)	44 356 (34.0)	116 256 (13.6)
2	29 455 (2.5)	6052 (6.5)	15 434 (11.8)	17 227 (2.0)
≥3	8797 (0.8)	2593 (2.8)	5306 (4.1)	4768 (0.6)
Insurance status				
Private	615 151 (52.8)	32 267 (34.9)	35 395 (27.1)	432 141 (50.7)
Medicaid	435 077 (37.3)	53 502 (57.8)	6029 (4.6)	17 371 (2.0)
Medicare	67 379 (5.8)	3215 (3.5)	83 790 (64.2)	364 414 (42.7)
Other government	11 343 (1.0)	673 (0.7)	1508 (1.2)	16 813 (2.0)
Not insured	20 751 (1.8)	1753 (1.9)	2223 (1.7)	11 225 (1.3)
Unknown	15 884 (1.4)	1084 (1.2)	1652 (1.3)	11 066 (1.3)
Income, $^a^				
≥63 000	437 449 (37.5)	30 176 (32.6)	37 973 (29.1)	306 582 (35.9)
48 999-62 999	311 632 (26.7)	24 704 (26.7)	35 532 (27.2)	227 446 (26.7)
38 000-47 999	243 629 (20.9)	21 744 (23.5)	32 398 (24.8)	185 032 (21.7)
<38 000	168 240 (14.4)	15 483 (16.7)	24 122 (18.5)	129 888 (15.2)
Unknown	4635 (0.4)	387 (0.4)	572 (0.4)	4082 (0.5)
Education^b^				
Highest	161 817 (13.9)	14 925 (16.1)	20 983 (16.1)	119 072 (14.0)
Intermediate-high	272 672 (23.4)	23 877 (25.8)	36 042 (27.6)	202 317 (23.7)
Intermediate-low	387 615 (33.3)	30 830 (33.3)	44 199 (33.8)	283 876 (33.3)
Lowest	339 314 (29.1)	22 526 (24.4)	28 859 (22.1)	244 179 (28.6)
Unknown	4167 (0.4)	336 (0.4)	514 (0.4)	3586 (0.4)
Facility type				
Academic	334 066 (28.7)	25 613 (28.1)	44 249 (33.9)	318 309 (37.3)
Nonacademic	774 900 (66.5)	65 415 (70.7)	86 055 (65.9)	534 195 (62.6)
Unknown	56 619 (4.9)	1466 (1.6)	293 (0.2)	526 (0.1)
Facility location				
Northeast	244 800 (21.0)	19 614 (21.2)	28 029 (21.5)	186 804 (21.9)
South	392 589 (33.7)	33 838 (36.6)	49 205 (37.7)	302 747 (35.5)
Midwest	289 346 (24.8)	24 217 (26.2)	36 497 (28.0)	232 691 (27.3)
West	182 231 (15.6)	13 359 (14.4)	16 573 (12.7)	130 262 (15.3)
Unknown	56 619 (4.9)	1466 (1.6)	293 (0.2)	526 (0.1)
Facility county				
Metropolitan	982 301 (84.3)	76 692 (82.9)	103 891 (79.6)	691 267 (81.0)
Urban	135 660 (11.6)	11 858 (12.8)	29 548 (15.7)	122 420 (14.4)
Rural	17 076 (1.5)	1611 (1.7)	2819 (2.2)	17 427 (2.0)
Unknown	30 548 (2.6)	2333 (2.5)	3339 (2.6)	21 916 (2.6)
AJCC clinical stage^c^				
0, low risk	52 000 (4.5)	NA	NA	237 739 (27.9)
I	715 138 (61.4)	52 975 (57.3)	105 266 (80.6)	378 034 (44.3)
II	319 411 (27.4)	24 388 (26.4)	25,31 (19.4)	237 257 (27.8)
III, high risk	79 036 (6.8)	15 131 (16.4)	NA	NA

Abbreviation: AJCC, American Joint Committee on Cancer; NA, not applicable; NCDB, National Cancer Database; NSC, non-small cell.

^a^ Income reflects the median income in the patient’s zip code.

^b^ Educational attainment reflects the number of adults in the zip code who did not graduate high school, with zip codes ranked in the US by tertile.

^c^ AJCC clinical prognostic group for breast cancer; Risk group (D’Amico classification) for prostate cancer (0 = low risk, 1 = intermediate risk, 2 = high risk).

**Table 2.** Descriptive Statistics by Cancer and Stage for Time-to-Treatment Initiation (TTI), NCDB 2004-2015

Cancer	TTI, median (IQR), d	Proportion treated by TTI, %			
		8-60 d	61-120 d	121-180 d	181-365 d
Breast					
Stage 0	34 (21-52)	81.6	15.4	2.2	0.8
Stage I	33 (22-48)	85.5	12.9	1.2	0.4
Stage II	33 (22-49)	84.9	12.8	1.5	0.8
Stage III	31 (21-46)	86.3	10.3	2.0	1.4
All stages	32 (21-48)	85.2	12.8	1.4	0.6
Colon					
Stage I	28 (18-43)	87.2	10.3	1.7	0.8
Stage II	23 (15-35)	92.9	6.0	0.8	0.3
Stage III	22 (14-34)	93.1	5.7	0.9	0.4
All stages	26 (16-40)	89.7	8.4	1.3	0.6
Non-small cell lung					
Stage I	41 (27-62)	73.3	21.6	3.4	1.4
Stage II	41 (27-60)	75.3	20.9	2.9	0.9
All stages	41 (27-62)	74.0	21.5	3.3	1.3
Prostate					
Low risk	83 (57-124)	28.0	46.0	16.1	10.0
Intermediate risk	77 (53-112)	32.8	46.0	14.2	7.1
High risk	80 (54-118)	31.8	44.3	16.2	7.7
All risks	79 (55-117)	31.2	45.5	15.3	8.1

Abbreviations: IQR, interquartile range; NCDB, National Cancer Database.

**Figure 2.**
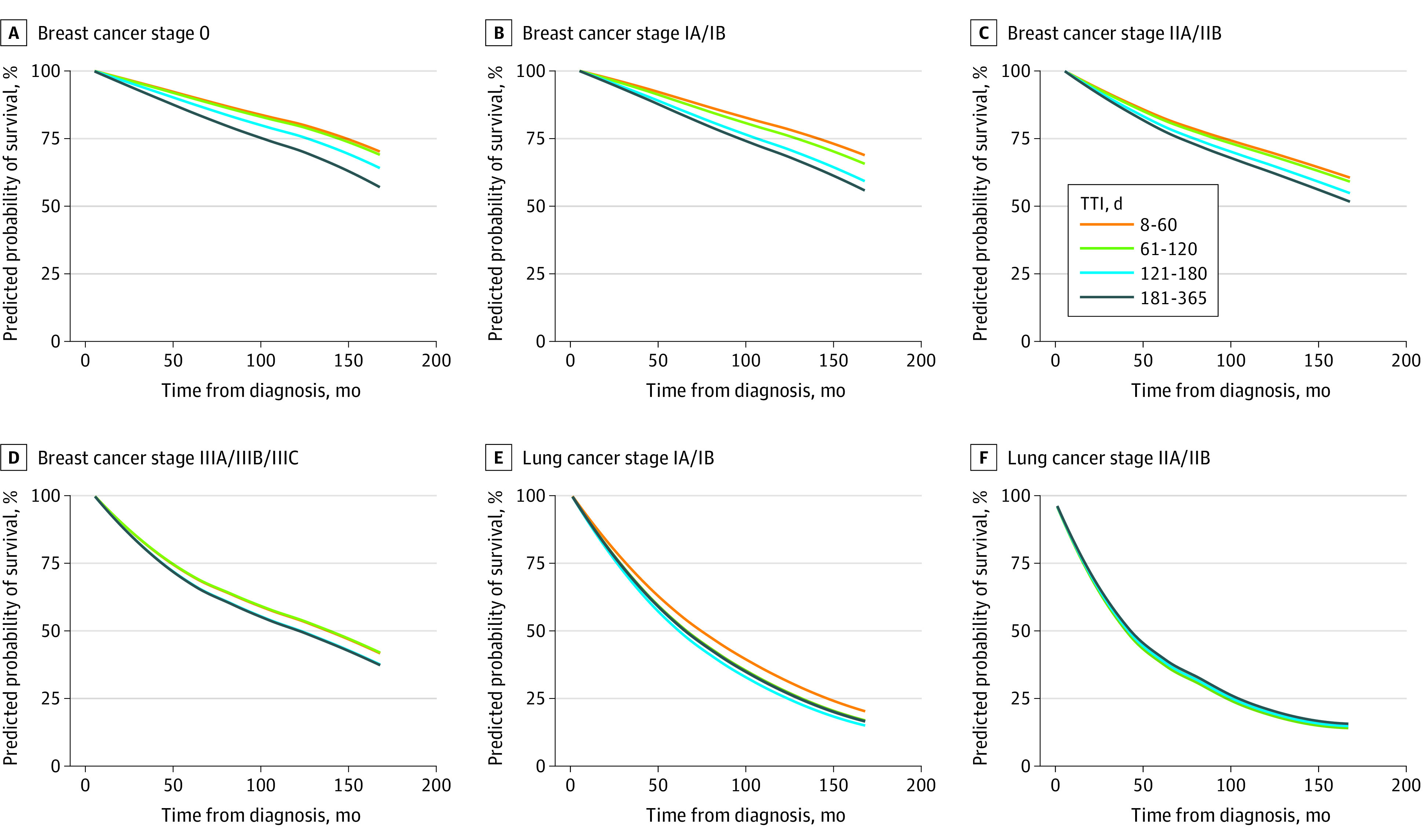
Predicted Probability of Survival of Breast and Non-Small Cell Lung Cancers from Diagnosis to Treatment Initiation

**Figure 3.**
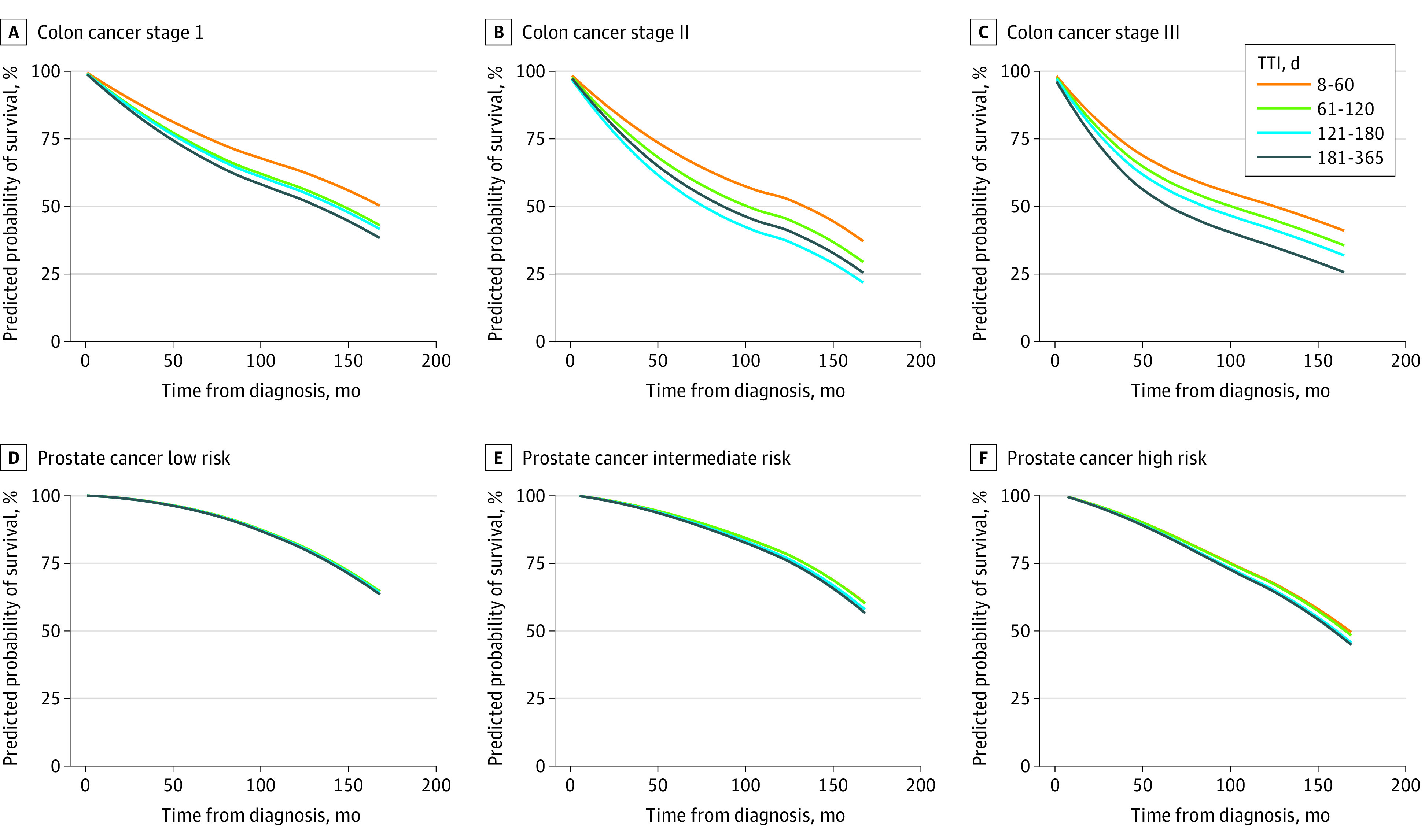
Predicted Probability of Survival of Colon and Prostate Cancers from Diagnosis to Treatment Initiation

The predicted 5-year and 10-year mortality rates were greatest for stage II NSCLC with TTI 61 to 120 days (62.0% and 81.2%, respectively). The predicted 5-year and 10-year mortality rates were lowest for low-risk prostate cancer patients treated within 60 days (4.9% and 17.3%, respectively). While analysis of all-stage prostate and breast did not reveal any association with delay, by-stage analysis demonstrated associations of high-risk and intermediate-risk prostate and stage I and II breast cancers with TTI of 61 to 120 days, and all-stage breast cancers with TTI greater than 121 days. Generally, increasing TTI and advanced stage and risk was associated with greater predicted mortality across all cancers (eg, predicted mortality: stage III breast cancer at TTI 181-365 d, 32.7% vs stage 0 breast cancer at TTI 8-60 d, 9.6%). Sensitivity analysis using 30-day intervals found no difference in trends by disease. Predicted 5-year and 10-year mortality by disease, stage, and TTI can be found in Table 3, with predicted mortality curves in Figures 2 and 3 (all differences significant except low-risk prostate cancer and stage II lung cancer). The most pronounced mortality association was for colon cancer (eg, 5 y predicted mortality, stage III: TTI 61-120 d, 38.9% vs. 181-365 d, 47.8%), followed by stage I NSCLC (5 y predicted mortality: TTI 61-120 d, 47.4% vs 181-365 d, 47.6%), while survival for prostate cancer was least associated (eg, 5 y predicted mortality, high risk: TTI 61-120 d, 12.8% vs 181-365 d, 14.1%), followed by breast cancer (eg, 5 y predicted mortality, stage I: TTI 61-120 d, 11.0% vs. 181-365 d, 15.2%). The interaction between TTI and race was not significant for most cancer stages tested.

**Table 3.** Predicted Mortality Table for 4 Common Forms of Cancer by Time to Definitive Treatment Initiation and Mortality Endpoint^a^

Cancer	Predicted mortality, 5 y, %				Predicted mortality, 10 y, %			
	TTI 8-60 d	TTI 61-120 d	TTI 121-180 d	TTI 181-365 d	TTI 8-60 d	TTI 61-120 d	TTI 121-180 d	TTI 181-365 d
Breast								
Stage 0	9.6	10.1	**12.1**	**15.3**	19.2	20.0	**23.6**	**28.9**
Stage I	9.7	**11.0**	**13.7**	**15.2**	20.7	**23.0**	**27.8**	**30.5**
Stage II	17.0	**17.7**	**20.0**	**21.7**	29.4	**30.5**	**34.0**	**36.5**
Stage III	29.6	29.4	**32.5**	**32.7**	45.4	45.2	**49.2**	**49.5**
Colon								
Stage I	22.1	**26.6**	**27.5**	**29.7**	36.3	**42.4**	**43.6**	**46.5**
Stage II	30.2	**36.2**	**43.2**	**39.7**	46.6	**54.0**	**62.1**	**58.1**
Stage III	34.6	**38.9**	**42.1**	**47.8**	48.9	**54.0**	**57.6**	**63.8**
Non-small cell lung								
Stage I	43.4	**47.4**	**49.6**	**47.6**	68.3	**72.6**	**74.8**	**72.8**
Stage II	60.8	62.0	60.6	59.6	80.1	81.2	80.0	79.1
Prostate								
Low risk	4.9	5.0	5.1	5.2	17.3	17.6	**17.9**	**18.3**
Intermediate risk	7.0	**7.4**	**7.9**	**8.3**	19.4	**20.4**	**21.8**	**22.6**
High risk	11.6	**12.8**	**13.8**	**14.1**	28.6	**31.2**	**33.2**	**33.8**

Abbreviation: TTI, time-to-treatment initiation.

^a^ Bolded cells contain projections of statistically significantly altered mortality for TTI when compared with the referent TTI interval of 8 to 60 days. For the cancer stages for which we identified an interaction between TTI and race, the displayed value is the overall effect size for all races.

Significance was found with TTI 8 to 60 days as referent only for stage III colon cancer at TTI 61 to 120 days (34.6% vs 38.9%; *P* = .03); intermediate-risk prostate cancer at TTI 121 to 180 days (7.0% vs 7.9%; *P* < .001); high-risk prostate cancer at TTI 181 to 365 days (11.6% vs. 12.8%; *P* = .02); and stage I breast cancer (predicted mortality vs TTI 8-60 days [9.7%]: 61-120 days, 11.0%; *P* = .04; 121-180 days, 13.7%; *P* = 0.01; 181-365 days, 15.2%; *P* = 0.03). In these subgroups all-cause mortality was higher for Black patients compared with White, but the effect size of increased TTI on mortality was less pronounced in Black patients. Race-specific predictions for 5-year and 10-year mortality by disease, stage, and TTI can be found in the eTable in the Supplement.

## Discussion

The COVID-19 pandemic has forced many hospitals in the US to grapple with decisions on how best to optimize allocation of limited health care resources. Postponing particular cancer treatments may lead to disease progression, metastasis, and, ultimately, cancer-related death. Guidelines developed by hospitals, states, and professional societies are largely based on expert opinion and often conflict with each other.^24,25,26,27^ Relying on the largest hospital-based registry in the US, we found varying associations between increased TTI and definitive treatment-eligible cancers, although at least some stages of all 4 of the most common cancers benefitted to some degree from a short TTI.

Of the various cancer stages examined, increased TTI was associated with the most negative survival effects for stage II and III breast cancer, in keeping with a recent systematic review finding that surgery should ideally occur less than 90 days from diagnosis and chemotherapy less than 120 days from diagnosis, with worse outcomes associated with delays for the disease stages and receptor statuses studied.^28^ Current recommendations by the COVID-19 Pandemic Breast Cancer Consortium advocate for neoadjuvant chemotherapy or endocrine therapy in patients who cannot undergo upfront surgery because of limited surgical capacity, although use of neoadjuvant systemic therapy in some of these populations (eg, ER-positive DCIS and T1N0 ER-positive cancers) remains unsubstantiated by high-level prospective data,^29^ with similar recommendations from the Society of Surgical Oncologists.^4,16^ The exception remains for patients with ER-negative DCIS, for whom no systemic treatment option exists. Our results showed significant differences in all-cause mortality with delayed definitive therapy even for stage I patients, although the effect size is clinically small. Patients with stage IA or IB breast cancer who were not treated until 61 to 120 days after diagnosis had 1.3% and 2.3% increased mortality at 5 years and 10 years, respectively, and those waiting longer suffered even greater increases in mortality. As such, our analysis underscores the importance of timely definitive treatment, even for stage I breast cancer.

The recommendations from both the American College of Surgeons and the Society of Surgical Oncologists for treatment of colon cancer during the COVID-19 pandemic are to continue to operate on known malignant masses, deferring treatment only for malignant polyps or sub–2 cm lung cancers.^4,16^ This urgency could be because colon cancer is subject to particularly long diagnostic intervals between first presentation for evaluation and date of diagnosis, which increases the total length of time between incidence of cancer and treatment.^30^ For colon cancer, we observed that each additional 60-day delay was associated with a 0.9% to 4.6% increase in 5-year all-cause mortality for stage I cancers, and a 3.2% to 6.0% increase in 5-year mortality for stage III cancers, with a longer 10-year time horizon resulting in larger effect sizes for increasing TTI. While our analyses may conflict with a systematic review finding no association with increased TTI on overall survival for colon cancer, this is partially because of differing methodology, as their longest delay category was greater than 55 days.^31^

For stage I lung cancer, we observed 4.0% to 6.2% absolute increases in 5-year mortality for increased TTI groups compared with the 8 to 60–day reference group, with larger effect sizes on 10-year mortality. The Kaplan-Meier survival curves for various levels of TTI for stage IIA/B cross at 2 separate time points, so we were unable to make conclusions about stage II lung cancer. The association between TTI and survival has previously been studied with conflicting results.^32^ Nadpara et al^33^ analyzed 16 747 patients in SEER and found a paradoxical association of improved survival with greater TTI, replicating prior work.^34^ Conversely, other studies have found better outcomes for timely treatment both in stage IA disease^35^ and in all localized disease.^36^ The COVID-19–related ACS recommendation is to treat all stage I and II patients except those with sub–2 cm masses.^16^ Meanwhile, the NCCN recommends deferral of stage IA1 tumors for 2 to 3 months, with urgent surgery within 1 month reserved for Stage IA2 through IIB tumors.^37^ Our analysis of increased treatment initiation in lung cancers therefore supports the current American College of Surgeons recommendations, but lack the granularity necessary to evaluate differentiating between stages IA1 and IA2-IIB per the NCCN guidelines.

As might be expected, increased TTI for prostate cancer was associated with lower mortality rates than for the other cancers examined. However, our findings for high-risk prostate cancer are of particular interest. Most prior work has found no association between clinical outcomes and delays in definitive therapy for localized prostate cancer, even with TTI of up to 6 months for high-risk prostate cancer.^38,39^ Based on these data, the NCCN recommended that treatment for low-risk and favorable intermediate-risk prostate cancer can be avoided during the COVID-19 pandemic, and unfavorable intermediate-risk and high-risk disease can safely be deferred for “upward of 6 months,”^24^ while other groups have recommended continued treatment of intermediate-risk and high-risk prostate cancer.^25,26,27^ By including low-risk prostate cancer patients in our analysis, we included a group that is widely considered safe to monitor and who should have a minimal and nonsignificant increase in mortality with treatment delay in the absence of upstaging,^20,39^ which indeed is what was observed. We found all-cause mortality differences of 2.2% at 5 years and 4.6% at 10 years between high-risk patients who were treated expeditiously vs those waiting 4 to 6 months (inside the current NCCN allowable deferral period), and differences of 0.9% at 5 years and 2.4% at 10 years for similar intermediate-risk patients. Our findings suggest that there is a limit to the length of treatment deferral that provides acceptable outcomes, and will be of use for urologists both in pandemic settings and otherwise.

Our study has important policy implications for the current pandemic, for triage of cancer care in resource-limited settings, and for future crises, as health care may be affected by future natural disasters, pandemics, and supply chain constraints. Our analyses suggest a rank order for prioritizing which patient cases could still be performed in resource-limited settings and the order in which cases should be added back to the treatment schedule as resource limitations ease. Our initial analysis of these common cancers provides foundational evidence on these discussions. Our study also found worse mortality for Black patients across cancers, but we found an inconsistent signal that increased TTI may have a weaker association with mortality for Black patients, potentially because of higher baseline risk.

### Limitations

Our study has several limitations. The main limitation is that drivers of increased TTIs in our patient cohort may differ from those in a pandemic. Patients in our cohort may have confounding factors which are linked to both worse outcomes and TTI, including low health literacy, poor access to care, and care at lower-quality hospitals, which could limit the generalizability of our findings.^9,10,11,19,22^ All patients are exposed to resource limitations in pandemic conditions, including those who are unaffected by these confounders, though disparities in care persist for racial minority groups.^40^ Therefore, our analysis can be thought of as a “worst case scenario” in estimating the effects of high times between diagnosis and treatment. We believe bias in this direction to be preferable, however, as it is more useful for hospital administrators, clinicians, and policymakers to know the upper limit effect size for predicted mortality potentially associated with triage decisions, especially when compared with underestimation owing to best-case biased analyses. We also note that in a pandemic specifically there are important other competing risks to consider, and the nuanced decision of whether the benefit of cancer treatment outweighs the risk of potentially lethal infection depends on factors outside the scope of our study such as comorbidities and local prevalence patterns.

We acknowledge that our mortality figures for NSCLC and colon cancer are higher than may be expected.^41^ However, we did include margin-positive patients by limiting our analysis to variables that can be determined during a triage process (margin status must be assessed postoperatively). We also report common NCDB-related limitations, including an inability to evaluate nonincluded outcomes (ie, cancer-specific survival) or to evaluate delayed diagnosis (because NCDB-inclusion requires cancer diagnosis). We cannot separate patients managed with watchful waiting who were treated after developing symptoms from those who delayed definitive therapy. We acknowledge that because of our convenience sample it is possible that nonsignificant findings were the result of a lack of power, although our samples were large. Finally, our cohort was limited to patients treated at facilities reporting to the NCDB, which may not accurately reflect national practice patterns, although more than 70% of cancer diagnoses are captured.^18^

## Conclusions

Examining delayed curative-intent treatment for breast, lung, colon, and prostate cancer, we found that all benefitted to some degree from a short interval between diagnosis and therapy. Some of our findings differ from current guideline recommendations. Specifically, our data support only limited deferral for prostate cancer, with length of deferral dependent on risk stratification. Future analyses will expand to other cancers, which may assist with treatment deferral decisions in resource-limited settings and provide a framework for prioritizing treatments as limitations ease.

## Notes

Supplement. **eAppendix 1.** Selection Criteria by Cancer Cohort **eAppendix 2.** Definitions of Definitive Therapy by Cancer **eTable.** Predicted Mortality Table by Time-to-Definitive-Treatment Initiation (TTI), Mortality Endpoint, Cancer, Stage, and Race
