# Supplementary material for: Assessment of Time-to-Treatment Initiation and Survival in a Cohort of Patients With Common Cancers
Source: JAMA Netw Open. 2020 Dec 14;3(12):e2030072. doi: 10.1001/jamanetworkopen.2020.30072 (PMC7737088; doi:10.1001/jamanetworkopen.2020.30072)
Supplement: Supplement. — eAppendix 1. Selection Criteria by Cancer Cohort eAppendix 2. Definitions of Definitive Therapy by Cancer eTable. Predicted Mortality Table by Time-to-Definitive-Treatment Initiation (TTI), Mortality Endpoint, Cancer, Stage, and Race [file jamanetwopen-e2030072-s001.pdf]

Supplemental Online Content

Cone EB, Marchese M, Paciotti M, et al. Assessment of time-to-treatment initiation and survival in a cohort of patients with common cancers. *JAMA Netw Open*. 2020;3(12):e2030072. doi:10.1001/jamanetworkopen.2020.30072

eAppendix 1: Selection Criteria by Cancer Cohort
eAppendix 2: Definitions of Definitive Therapy by Cancer
eTable. Predicted Mortality Table by Time to Definitive Treatment Initiation (TTI), Mortality Endpoint, Cancer, Stage, and Race

This supplemental material has been provided by the authors to give readers additional information about their work.

© 2020 Cone EB et al. *JAMA Network Open.*

eAppendix 1: Selection criteria by cancer cohort.

For all cancers we used clinical staging. For all the tumors, patients with metastases, patients with unknown staging/risk groups, and patients with missing follow-up data were excluded.

For breast cancer, we included stage 0, I (IA, IB), II (IIA, IIB), III (IIIA, IIIB, IIIC) according to the American Joint Committee on Cancer (AJCC) prognostic clinical stage group (8th edition). This risk classification takes into account TNM stage, grade of the tumor, receptor (Estrogen Receptor, Progesterone Receptor, HER2) status. We excluded inflammatory breast cancer. For prostate cancer, we included low-, intermediate- and high-risk prostate cancer according to the D'Amico classification, a widely used risk classification schema amongst urologists incorporating stage, grade, and PSA.[45] We excluded clinical T4, and clinical N positive patients, given that the optimal local treatment for these patients remains controversial. For NSCLC, we included stage IA, IB, IIA, IIB. Stages were categorized as: I (IA and IB) and II (IIA and IIB). For purpose of this study, we excluded stage 0 NSCLC. We excluded stage III patients as the NCDB lacked crucial variables such as performance status to determine appropriate treatment. We excluded histology other than adenocarcinoma or squamous cell, which accounted for more than 80% of the NSCLC. For colon cancer, we included stage I, II (IIA, IIB, IIC), III (IIIA, IIIB, IIIC). We excluded clinical stage 0 as most patients are adequately treated during diagnosis via polyp removal so there is no separate

© 2020 Cone EB et al. *JAMA Network Open.*

treatment episode, and also excluded histology other than adenocarcinoma and primary appendix cancers.

© 2020 Cone EB et al. *JAMA Network Open.*

eAppendix 2: Definitions of definitive therapy by cancer

For breast cancer, definitive therapy was defined as surgery or neoadjuvant systemic therapy followed by surgery. For prostate cancer, definitive therapy was defined as surgery or some form of radiotherapy. We included low risk prostate cancer in the analysis as a comparator group that does not necessitate active treatment, as active surveillance is an accepted option that should have a minimal increase in mortality with treatment delay in the absence of upstaging.[24] For non-small cell lung cancer, definitive therapy was defined as surgery, neoadjuvant chemotherapy followed by surgery, chemoradiation, or radiation. For colon cancer, definitive therapy was defined as surgery.

Patients who did not receive any definitive therapy were excluded. Other therapies which excluded patients from the cohort included local tumor destruction (lack of pathology specimen) or excision (excisional biopsy, polypectomy, etc.), and cases where contiguous organ resection was performed (pelvic exenteration). For breast, patients receiving radiotherapy as first treatment were excluded, as well as patients who did not undergo surgery. For prostate, transurethral resection, subtotal, segmental, or simple prostatectomy were excluded. For colon, patients receiving systemic therapy as first treatment were excluded, since neoadjuvant chemotherapy was not a standard of care option for resectable colon cancer prior to 2016.

© 2020 Cone EB et al. *JAMA Network Open.*

eTable 1. Predicted mortality table by time to definitive treatment initiation (TTI), mortality endpoint, cancer, stage, and race. Table values represent the projected 5- or 10-year mortality probability (%) for that TTI interval, cancer, stage, and race. Shaded cells had an interaction detected between TTI and race for the mortality projection (reference 8-60 days TTI).

	**Predicted mortality (%)**		**5 years**				**10 years**			
	**Time to treatment initiation**		**8-60 days**	**61-120 days**	**121-180 days**	**181-365 days**	**8-60 days**	**61-120 days**	**121-180 days**	**181-365 days**
**Breast**	**Stage 0**	**White**	9.6	10.1	12.1	15.3	19.2	20.1	23.6	28.9
		**Black**	10.6	11.1	13.3	16.7	21.0	21.9	25.7	31.3
	**Stage I**	**White**	9.8	**14.1**	**15.8**	**11.2**	20.6	**28.4**	**31.2**	**23.1**
		**Black**	11.2	**12.0**	**13.8**	**14.3**	24.4	**26.0**	**29.4**	**30.3**
	**Stage II**	**White**	16.8	17.6	19.8	21.4	29.1	30.3	33.7	36.2
		**Black**	19.4	20.2	22.7	24.6	33.2	34.4	38.1	40.9
	**Stage III**	**White**	28.8	28.6	31.7	31.9	44.7	44.4	48.5	48.7
		**Black**	34.1	33.9	37.2	37.4	51.6	51.3	55.6	55.9
**Colon**	**Stage I**	**White**	22.2	26.7	27.6	29.8	36.5	42.6	43.8	46.7
		**Black**	23.0	27.6	28.5	30.8	37.6	43.8	45.0	48.0
	**Stage II**	**White**	30.3	36.3	43.3	39.8	46.7	54.2	62.3	58.3
		**Black**	31.5	37.7	44.9	41.2	48.3	55.8	63.9	59.9
	**Stage III**	**White**	35.3	**41.4**	44.8	47.6	49.4	**56.4**	60.2	63.1
		**Black**	34.1	**33.8**	38.0	58.7	51.4	**51.0**	56.2	78.2

© 2020 Cone EB et al. *JAMA Network Open.*

**Non-small cell lung**	**Stage I**	**White**	43.3	47.1	49.5	47.6	67.7	71.7	74.1	72.1
		**Black**	41.9	45.6	48.0	46.1	66.1	70.1	72.6	70.6
	**Stage II**	**White**	60.4	61.8	60.7	59.6	79.7	80.9	80.1	79.1
		**Black**	59.4	60.9	59.8	58.7	78.9	80.2	79.3	78.3
**Prostate**	**Low risk**	**White**	5.0	4.9	5.0	5.1	17.7	17.4	17.7	18.1
		**Black**	5.1	6.0	6.1	6.2	18.1	20.8	21.2	21.6
	**Intermediate risk**	**White**	6.9	7.3	**7.9**	8.1	19.4	20.5	**22.1**	22.6
		**Black**	8.5	8.6	**8.6**	9.7	22.4	22.5	**22.6**	25.1
	**High risk**	**White**	11.7	13.0	14.0	**14.4**	28.7	31.4	33.4	**34.3**
		**Black**	12.1	12.9	13.9	**13.5**	30.0	31.7	33.8	**32.9**

© 2020 Cone EB et al. *JAMA Network Open.*
